# The Prevalence of Scabies and Impetigo in the Solomon Islands: A Population-Based Survey

**DOI:** 10.1371/journal.pntd.0004803

**Published:** 2016-06-27

**Authors:** Daniel S. Mason, Michael Marks, Oliver Sokana, Anthony W. Solomon, David C. Mabey, Lucia Romani, John Kaldor, Andrew C. Steer, Daniel Engelman

**Affiliations:** 1 Centre for International Child Health, University of Melbourne, Melbourne, Australia; 2 Clinical Research Department, London School of Hygiene and Tropical Medicine, London, United Kingdom; 3 Hospital for Tropical Diseases, London, United Kingdom; 4 Ministry of Health, Honiara, Solomon Islands; 5 Department of Control of Neglected Tropical Diseases, World Health Organization, Geneva, Switzerland; 6 Kirby Institute, University of New South Wales, Sydney, Australia; 7 Group A Streptococcal Research Group, Murdoch Childrens Research Institute, Melbourne, Australia; University of California San Diego School of Medicine, UNITED STATES

## Abstract

**Background:**

Scabies and impetigo are common, important and treatable skin conditions. Reports from several Pacific island countries show extremely high prevalence of these two conditions, but for many countries, including the Solomon Islands, there is a paucity of epidemiological data.

**Methodology:**

Ten rural villages in the Western Province of the Solomon Islands were included in the study, chosen so that data collection could be integrated with an existing project investigating clinical and serological markers of yaws. All residents were eligible to participate, and 1908 people were enrolled. Participants were interviewed and examined by a paediatric registrar, who recorded relevant demographic information, and made a clinical diagnosis of scabies and/or impetigo, severity and distribution.

**Principal Findings:**

The total unweighted prevalence of scabies was 19.2% (95% confidence interval [CI] 17.5–21.0), and age and gender weighted prevalence 19.2% (95%CI 16.7–21.9). The adult prevalence of scabies was 10.4% (95%CI 8.2–13.2), and the highest prevalence was found in infants < 1 year of age (34.1%, adjusted odds ratio [AOR] compared with adults: 3.6, 95%CI 2.2–6.0) and children aged 1–4 years (25.7%, AOR 2.6, 95%CI 1.7–3.9). Scabies affected two or more body regions in 80.9% of participants, and 4.4% of scabies cases were classified as severe. The total unweighted prevalence of active impetigo was 32.7% (95%CI 30.6–34.8), and age and gender weighted prevalence 26.7% (95%CI 24.2–29.5). The highest prevalence was found in children aged 1–4 years (42.6%, AOR compared with adults: 4.1, 95%CI 2.9–5.8). Scabies infestation was associated with active impetigo infection (AOR 2.0, 95%CI 1.6–2.6); with 41.1% of active impetigo cases also having scabies.

**Conclusions and Significance:**

Scabies and impetigo are very common in the rural Western Province of the Solomon Islands. Scabies infestation is strongly associated with impetigo. Community control strategies for scabies may reduce the burden of both conditions and their downstream complications.

## Author Summary

Scabies, a parasitic infection, and impetigo, a superficial bacterial infection, are treatable skin conditions found most commonly in resource-limited settings. Scabies is strongly associated with impetigo. Complications of impetigo include sepsis and invasive infections, and post-infective complications such as acute post streptococcal glomerulonephritis and acute rheumatic fever. Good data on scabies and impetigo prevalence are lacking for most countries, but existing evidence suggests the Pacific region has among the highest prevalence of these conditions in the world. Our study aimed to establish the prevalence of scabies and impetigo in the Western Province of the Solomon Islands, a South Pacific nation made up of over 900 islands and over 500,000 inhabitants. We assessed over 1900 people of all ages, and found a very high burden of skin infections, with scabies affecting one in five people, and active impetigo in one in three. Infants and children were affected more than adults. Scabies infestation was strongly associated with impetigo, supporting the hypothesis that community control strategies for scabies may be successful in reducing the burden of impetigo and its sequelae.

## Introduction

Scabies is a parasitic infestation of the skin by the mite *Sarcoptes scabiei* var *hominis*. Cutaneous manifestations including papules, burrows and intense pruritus are mediated through host immune responses to mite products [1]. Transmission is common between family members, sexual partners, and within institutional settings [2]. Impetigo is a superficial bacterial skin infection caused primarily by *Streptococcus pyogenes* and *Staphylococcus aureus*, and has been associated with scabies infestation in tropical environments [3, 4]. Suppurative complications of impetigo include cellulitis, abscesses, septic arthritis, osteomyelitis and septicaemia. Streptococcal skin infection has been implicated in up to 50% of cases of acute post streptococcal glomerulonephritis in tropical settings [5], and contributes to chronic kidney disease in adulthood [6]. There is also strong epidemiological data linking streptococcal impetigo with acute rheumatic fever and rheumatic heart disease, which causes significant morbidity and mortality worldwide [7].

Although there is lack of good quality epidemiological data on the occurrence of scabies and impetigo, it appears that the greatest burden of these conditions is in settings with hot, humid climates, with the highest prevalence found in young children [8]. Multiple studies have shown increased scabies prevalence associated with poverty and overcrowding, especially within sleeping quarters [9, 10]. It has been estimated that more than 100 million people are affected by scabies worldwide at any time [11].

Despite the Pacific region having some of the highest reported prevalence figures of scabies and impetigo in the world [8], there is a paucity of data from the Solomon Islands. The aim of this study was to establish the prevalence of scabies and active impetigo in remote communities of the Western Province of the Solomon Islands, and to investigate the clinical features of disease and epidemiologic associations in this setting.

## Methods

### Setting

The Solomon Islands is a nation of 992 islands in the South Pacific, comprising large volcanic islands and low lying coral atolls. The Solomon Islands ranked 157^th^ on the United Nations Human Development Index (HDI) in 2014 [12], and has considerably poorer health outcomes compared to South Pacific regional averages [13]. The population of approximately 516,000 comprises predominantly Melanesian peoples (95.3%), of whom 41% are aged below 15 years. Over 80% of the population live in rural areas without access to electricity or formal waste disposal services [14].

### Study Design, Sampling and Procedures

**Fig 1:**
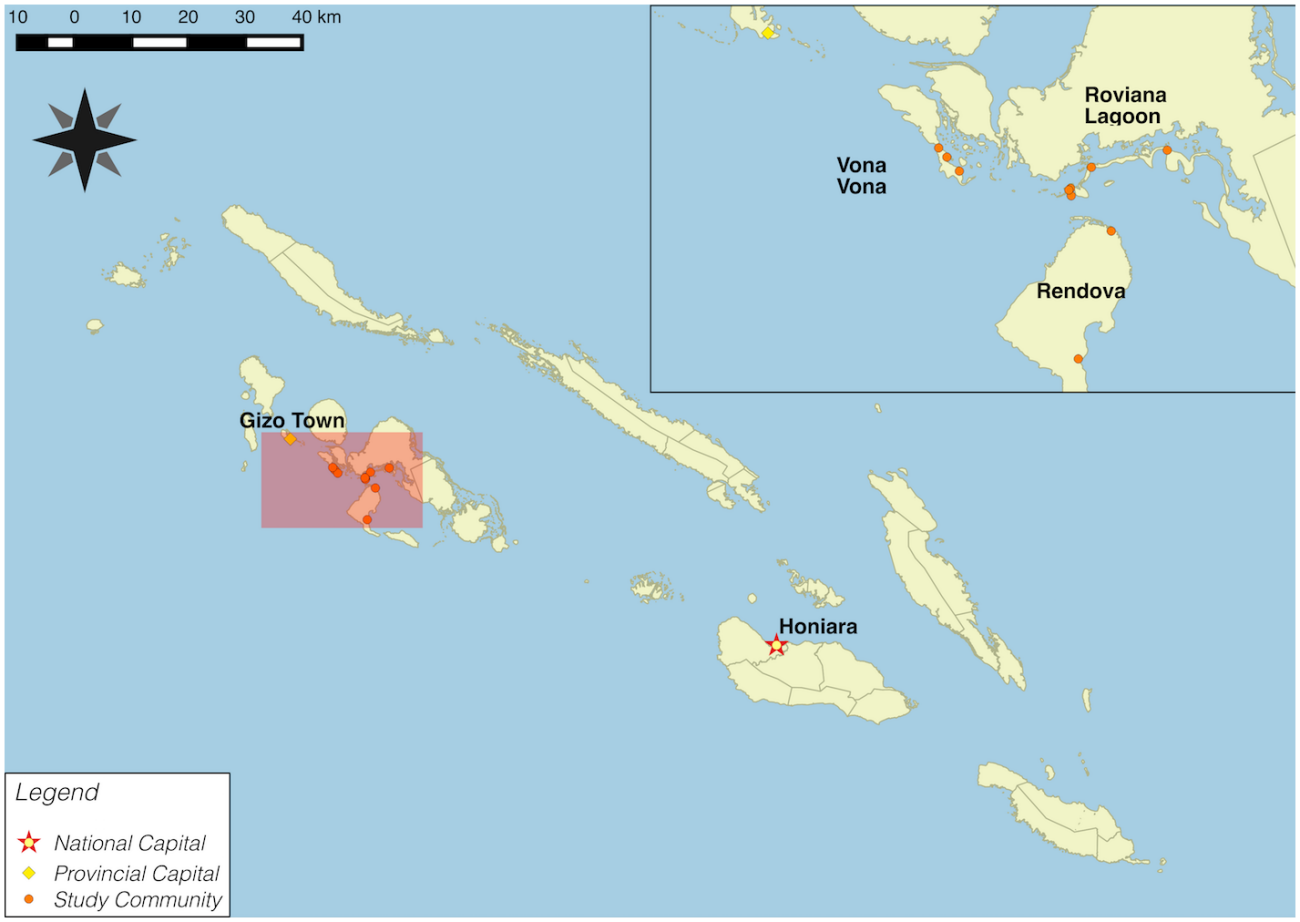
Map outlining distinct study regions within the Western Province of the Solomon Islands: Vona vona Lagoon, Roviana Lagoon and Rendova.

We undertook a cross-sectional population-based study in the Western Province of the Solomon Islands during November 2014. This was undertaken in ten villages within three distinct geographical regions within the Western Province (Vona Vona Lagoon, Roviana Lagoon and Rendova, Fig 1). Dwellings in this region are typically made of local wood. Although variable in size and structure, most comprise one to two sleeping quarters for all house inhabitants.

For logistical reasons data collection was integrated with an existing project investigating clinical and serological markers of yaws [15], with selected villages having participating in a mass drug administration for trachoma using azithromycin, a drug that is also active against yaws.

Ethics approval for the study was obtained from the London School of Hygiene and Tropical Medicine (LSHTM Ref 6358) and the National Health Research and Ethics Committee of the Solomon Islands Ministry of Health and Medical Services (HRC14/27). Both of these committee’s approved the research study protocols and methodology.

Following permission from community leaders, a public meeting was arranged for all village members where the study procedures and objectives were explained. Written consent was obtained for all participants. Guardians provided consent for participants aged below 18 years. All community members were eligible for this study, which took place at a pre-arranged public place.

All participants were interviewed and examined by a doctor. Assessments were conducted in the local medical clinic where one existed, or an appropriate community space. The doctor was a paediatric trainee registrar who had received specific training on the diagnosis of scabies and impetigo prior to the study, in addition to 18 months of clinical experience in tropical regions of northern Australia. Clinical history was taken in a combination of English and Solomon Island Pijin. Local nursing staff assisted with translation to local dialects (Roviana and Touo) when required. Demographic information including gender, age, and number of household and bedroom inhabitants was recorded.

Examination of the skin focused on bodily regions most commonly affected by scabies and impetigo. For infants and young children, the whole body was fully examined. For older children and adults, examination of sensitive areas such as the groin, buttocks, breasts and torso was only undertaken if there was adequate privacy. All participants who had scabies clinically on history and/or physical examination were asked whether they had similar lesions in these regions. The clinical diagnosis of scabies was based on features including morphology (burrows, papules, nodules, vesicles) and body distribution of rash; presence of pruritus on history or clinical examination evidence of excoriation; contact history with individuals with a similar rash and itch; and consideration of differential diagnoses [1]. The distribution of scabies lesions was noted using nine pre-defined body regions. Following other studies in the Pacific, scabies severity was classified by the number of lesion present as mild (≤10 lesions), moderate (11–49 lesions) or severe (≥ 50 lesions or crusted scabies) [3].

Active impetigo was diagnosed on the basis of discrete papular, pustular or ulcerative lesions with associated erythema, crusting, bullae or frank pus. Inactive impetigo was diagnosed by the presence of discrete, non-confluent healed superficial skin lesions. Severity of active impetigo was classified as very mild (≤ 5 lesions), mild (6–10 lesions), moderate (11–49 lesions) or severe (≥ 50 lesions) [3]. Participants diagnosed with any condition were counselled regarding the diagnosis and provided with an information sheet and referral letter to the nearest medical clinic for treatment according to standard local protocols [16].

### Statistical Analysis

Data were recorded on an Android smartphone, using a customized electronic form created using Open Data Kit (ODK, Seattle, USA, 2010). Individual data collection forms were uploaded from the phones to an encrypted, password protected private server using ODK Aggregate. Data were analysed using Stata version 13.1 (StataCorp, Texas, USA) and Microsoft Excel 2011 version 14.4.8 (Microsoft Corporation, Washington, USA). The prevalences of scabies and impetigo were directly adjusted for gender and age using our sample demographic data. Adjusted odds ratios (AOR) for scabies and impetigo prevalence were calculated using a multivariate logistic regression model that incorporated the demographic factors of gender, age group, number of people living in house and number of people sleeping in the same room. Attributable risk for scabies as a cause of impetigo in our sample was calculated using standard methods [17].

## Results

We enrolled 1908 participants, representing 49% of the census-listed population of the communities visited, and 2.5% of the total population of the Western Province. There was an overrepresentation of female participants and children aged less than 15 years within the sample (Table 1).

**Table 1:** Demographic characteristics of sample compared to Western Province and national census populations.

	Study Sample (N = 1908)	Census Western Province (N = 76,649)	Census Solomon Islands (N = 515,870)
	n (%)	n (%)	n (%)
**Gender**			
**Male**	805 (42.2)	39,926 (52.1)	264,455 (51.3)
**Female**	1103 (57.8)	36723 (47.9)	251415 (48.7)
**Age (years)**			
**0–4**	337 (17.7)	11,082 (14.5)	76,500 (14.8)
**5–14**	837 (43.9)	19,601 (25.6)	132,963 (25.8)
**15–24**	148 (7.8)	13,962 (18.2)	96,542 (18.7)
**≥ 25**	586 (30.7)	32,004 (41.8)	209,865 (40.7)
**Mean People per household (SD)**	6.1 (2.5)	5.3 (0.3)	5.5 (0.6)

The total unweighted prevalence of scabies was 19.2% (95% confidence interval [95%CI] 17.5–21.0, Table 2). The adjusted prevalence, weighted for gender and age was 19.2% (95%CI 16.7–21.9). The prevalence was higher in males than females, (prevalence 24.1% vs 15.6%, adjusted odds ratio [AOR] 1.4, 95%CI 1.1–1.8, Table 2), and in children than adults, most notably in infants (prevalence 34.1% vs 10.4%, AOR 3.6, 95%CI 2.2–6.0). Compared to participants with 5 or less household members there was a modest but statistically significant increase in the prevalence of scabies in participants living with six to ten people per household (AOR 1.4, 95%CI 1.1–1.7), but not for participants living with more than ten people per household (AOR 1.2, 95%CI 0.7–2.0, *p* = 0.61) or with more than two people per room. Scabies was classified as moderate in 47.8% and severe in 4.4% of cases. No cases of confirmed crusted scabies were identified.

**Table 2:** Scabies prevalence and severity (AOR: adjusted odds ratio; CI: confidence interval).

	Study Sample (N = 1908)	No. with Scabies	AOR (95% CI)	Severity		
	n	n (%)		Mild n (%)	Moderate n (%)	Severe n (%)
**Gender**						
**Male**	805	194 (24.1)	1.4 (1.1–1.8)	86 (44.3)	97 (50)	11 (5.7)
**Female**	1103	172 (15.6)	1.0	89 (51.7)	78 (45.3)	5 (2.9)
**Age (y)**						
**<1**	41	14 (34.1)	3.6 (2.2–6.0)	2 (14.3)	8 (57.1)	4 (28.6)
**1–4**	296	76 (25.7)	2.6 (1.7–3.9)	33 (43.4)	41 (54)	2 (2.6)
**5–14**	837	190 (22.7)	2.3 (1.6–3.1)	103 (54.2)	80 (42.1)	7 (3.7)
**15–24**	148	25 (16.9)	1.7 (1.0–2.9)	11 (44)	12 (48)	2 (8)
**≥25**	586	61 (10.4)	1.0	26 (42.6)	34 (55.7)	1 (1.6)
**Total**	1908	366 (19.2)		175 (47.8)	175 (47.8)	16 (4.4)

**Fig 2:**
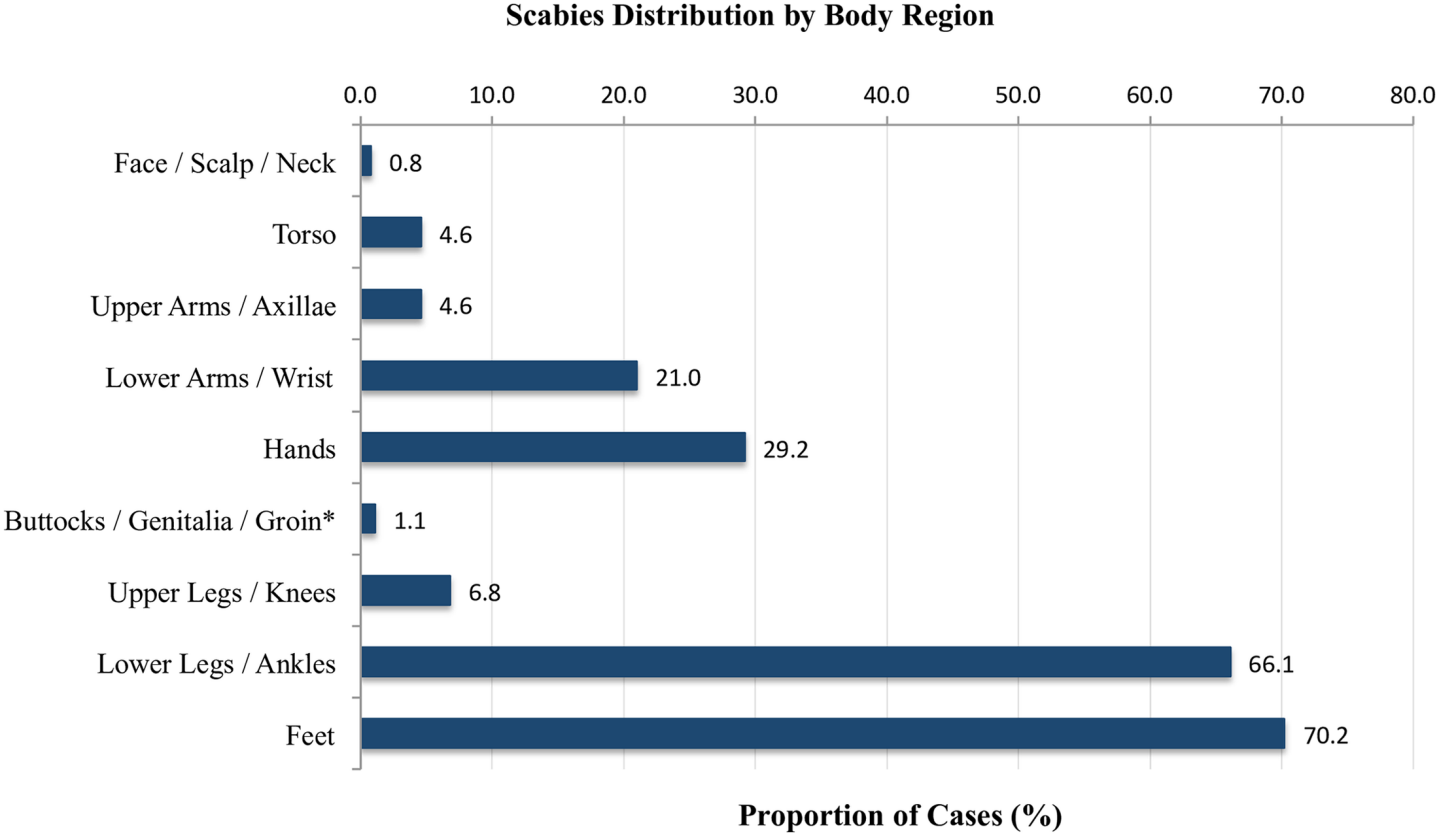
Scabies distribution by body region. *These regions were not routinely examined in all participants.

Scabies most commonly affected lower legs, ankles, feet, wrists and hands (Table 3, Fig 2). In our study 80.9% of participants with scabies had more than one body region involved. The clinical pattern in infants was more likely to involve the head and neck (14.3% of infants compared to 0% of adults) and torso (50% of infants compared to 1.6% of adults, OR 31.3). Infants were also more likely to have more than one body region involved (92.9% of infants compared to 80.2% of adults, OR 1.2).

**Table 3:** Rash distribution of scabies cases (body regions are not mutually exclusive).

Body region involved	Age (years)					Total
	<1 n (%)	1–4 n (%)	5–14 n (%)	15–24 n (%)	≥25 n (%)	n (%)
**Face / Scalp / Neck**	2 (14.3)	1 (1.3)	0	0	0	3 (0.8)
**Torso**	7 (50.0)	4 (5.3)	5 (2.6)	0	1 (1.6)	17 (4.6)
**Upper Arms / Axillae**	3 (21.4)	3 (3.9)	5 (2.6)	1 (4)	5 (8.2)	17 (4.6)
**Lower Arms / Wrist**	6 (42.9)	18 (23.7)	43 (22.6)	6 (24)	4 (6.6)	77 (21.0)
**Hands**	10 (71.4)	23 (30.3)	59 (31.1)	6 (24)	9 (24.6)	107 (29.2)
Buttocks / Genitalia / Groin*	2 (14.3)	0	2 (1.1)	0	0	4 (1.1)
**Upper Legs / Knees**	4 (28.6)	9 (11.8)	9 (4.7)	2 (8)	1 (1.6)	25 (6.8)
**Lower Legs / Ankle**	7 (50.0)	54 (71.1)	121 (63.7)	18 (72)	42 (68.9)	242 (66.1)
**Feet**	7 (50.0)	57 (75)	130 (68.4)	18 (72)	45 (73.8)	257 (70.2)
**Total cases**	14	76	190	25	61	366

*These body regions were not routinely examined (see Discussion)

The total unweighted prevalence of impetigo was very high at 87.4% (95%CI 85.6–88.8) of participants, including active impetigo (32.7%, 95%CI 30.6–34.8); inactive impetigo (83.2%, 95%CI 81.4–84.8) or both (28.4%, 95%CI 26.4–30.5).

The adjusted prevalence of active impetigo, weighted for gender and age was 26.7% (95%CI 24.2–29.5). The prevalence of active impetigo was higher in males than females (prevalence 43.7%, AOR 1.9, 95%CI 1.5–2.4) and in children than adults, particularly children aged one to four years (prevalence 42.6%, AOR 4.1, 95%CI 2.9–5.8, Table 4). Of those with active impetigo, 90% had very mild or mild disease. There was no statistically significant association between active impetigo prevalence and the number of people per household.

**Table 4:** Impetigo prevalence and severity (AOR: adjusted odds ratio; CI: confidence interval).

	Study Sample (N = 1908)	No. with Active Impetigo (%)	AOR (95% CI)	Severity			
	n	n (%)		Very mild n (%)	Mild n (%)	Moderate n (%)	Severe n (%)
**Gender**							
**Male**	805	352 (43.7)	**1.9** (1.5–2.4)	196 (55.7)	118 (33.5)	35 (9.9)	3 (0.9)
**Female**	1103	271 (24.6)	**1.0**	166 (61.3)	81 (29.9)	24 (8.9)	0
**Age (y)**							
**<1**	41	3 (7.3)	**1.2** (0.7–2.0)	0	1 (33.3)	1 (33.3)	1 (33.3)
**1–4**	296	126 (42.6)	**4.1** (2.9–5.8)	67 (53.2)	43 (34.1)	16 (12.7)	0
**5–14**	837	378 (45.2)	**4.0** (3.1–5.3)	217 (57.4)	124 (32.8)	35 (9.3)	2 (0.5)
**15–24**	148	29 (19.6)	**1.4** (0.8–2.2)	14 (48.3)	12 (41.4)	3 (10.3)	0
**≥25**	586	87 (14.8)	**1.0**	64 (73.6)	19 (21.8)	4 (4.6)	0
**Total**	1908	623 (32.7)		362 (58.1)	199 (31.9)	59 (9.5)	3 (0.5)

The prevalence of active impetigo was higher in those with scabies (179 of 366, 48.9%, AOR 2.0, 95%CI 1.6–2.6) compared to those without scabies (444 of 1542, 28.8%), corresponding to an attributable risk of scabies as a cause of impetigo of 41.1% (95%CI 32.9–48.3). When compared to participants without scabies, impetigo prevalence was higher in those with moderate *scabiegs* (prevalence 57%, AOR 3.1, 95%CI 2.2–4.4) and with severe scabies (prevalence 62.5%; AOR 4.1, 95%CI 1.4–12.2).

## Discussion

In this cross-sectional survey we report a substantial prevalence of scabies and impetigo in ten villages of the Western Province of the Solomon Islands. The age and gender adjusted prevalences of scabies (19%) and impetigo (27%) are lower than the 25% and 40% respectively in a 1997 study from five islands in the Malaita Province in the Solomon Islands [18]. Our results are also similar to other studies in the Pacific region, including Fiji [3, 18–21], Vanuatu [22], Timor-Leste [23] and indigenous communities of northern Australia [9, 24].

Clinical findings and epidemiological associations supported findings from neighboring Pacific island countries. The prevalence of scabies was highest in infants and pre-school aged children, suggesting that these age groups are important in continued community transmission [20]. There was a strong association between the presence of scabies infestation and active impetigo, suggesting that scabies infestation is an important risk factor for bacterial infection of the skin. This association is well supported by previous data from the Pacific region, including multiple studies from Fiji [3, 20]. We did not observe a consistent association between scabies or impetigo prevalence and measures of household crowding.

The clinical distribution of scabies, in all groups but infants, followed the classic acral distribution previously described [1]. However, our finding that the lower extremities were affected more frequently than the upper limbs is in contrast to some other studies [3]. One possible explanation is a lack of footwear and clothing covering the ankles and feet in the communities we examined. Similar to previous studies, we observed that scabies in infants has a more varied presentation, with rash more commonly affecting the head and multiple body regions [25].

Our study has several limitations. Firstly participation was estimated at 49% of communities visited, and compared to the national population, the sample overrepresented females and children aged less than 15 years. Non participation among the eligible target population was due primarily to community members not being present in the village at the time of study. A small number consented but did not wait to be examined. We were able to adjust our analysis for age and gender, but cannot be certain of the influence of only collecting data from half of the population. Second, due to available facilities and infrastructure, clinical examination was often undertaken with limited privacy for participants. In this context, examination of all body regions was frequently not appropriate, and therefore data on severity and distribution of disease in some body areas is likely underestimated. This was particularly relevant for the buttocks/genitalia/groin region. Third, the diagnosis of scabies and impetigo was made on the basis of directed clinical history and skin examination alone. Skin scrapings for direct microscopy and magnification with dermoscopy were not used, and may have enhanced accuracy. Diagnostic accuracy is dependent upon the clinical acumen and experience of the examiner. The examining doctor, whilst having relevant clinical experience, did not have formal training in tropical dermatology, which may have led to some diagnostic misclassification. However, our prevalence estimates are consistent with comparable studies, suggesting that this was unlikely to be a significant factor. Further development of standardized clinical criteria and algorithms for the diagnosis of scabies and impetigo in field settings [26] would facilitate more accurate data collection and comparisons of global epidemiological data. Finally, all communities selected for the study had participated in a trachoma control program, which included community administration of a single dose of azithromycin in June 2014. As azithromycin has activity against *S*. *pyogenes* and *S*. *aureus*, this may have reduced the burden of impetigo observed. Limited previous data has supported this hypothesis [27], however further investigation into the effect of single dose azithromycin against staphylococcal and streptococcal skin disease is warranted.

Despite these limitations, there are important clinical and public health implications arising from our study. Scabies and impetigo are both very common diseases in the Western Province of the Solomon Islands, with scabies and/or impetigo affecting 42.5% of our study participants and an even greater proportion of children. These conditions place a great burden on these communities, with clinical management of individual cases consuming a significant proportion of limited health resources. Scabies appears to be a significant risk factor for the development of active impetigo. Therefore, if public health programs are to reduce the burden of impetigo, its downstream complications and associated morbidity, there is a need to explore methods for community control of scabies. Studies of mass drug administration using both topical scabicides and oral ivermectin in the Solomon Islands [18], Fiji [21] and other countries appear highly promising in reducing scabies and impetigo prevalence, and require serious consideration for ongoing management of these neglected tropical diseases of the skin.

## Supporting Information

S1 Checklist STROBE checklist. Scabies & Impetigo in Solomon Islands
(DOC)

S1 Dataset Raw data from all 1908 participants. (XLSX)

S2 Dataset Data for number of people living in each house and number of people sleeping in each room. (XLSX)
